# Post-Traumatic Stress Symptoms in Healthcare Workers Dealing with the COVID-19 Pandemic: A Systematic Review

**DOI:** 10.3390/ijerph18020601

**Published:** 2021-01-12

**Authors:** Gabriele d’Ettorre, Giancarlo Ceccarelli, Letizia Santinelli, Paolo Vassalini, Giuseppe Pietro Innocenti, Francesco Alessandri, Alexia E. Koukopoulos, Alessandro Russo, Gabriella d’Ettorre, Lorenzo Tarsitani

**Affiliations:** 1 Department of Occupational Medicine, Local Health Authority of Lecce, 73100 Lecce, Italy; gabriele.det@libero.it; 2 Department of Public Health and Infectious Diseases, Sapienza University of Rome, 00161 Rome, Italy; giancarlo.ceccarelli@uniroma1.it (G.C.); letizia.santinelli@uniroma1.it (L.S.); paolo.vassalini@uniroma1.it (P.V.); giuseppepietro.innocenti@uniroma1.it (G.P.I.); alessandro.russo1982@gmail.com (A.R.); 3 Department of Anesthesia and Intensive Care Medicine, Sapienza University of Rome, 00161 Rome, Italy; francesco.alessandri@uniroma1.it; 4 Department of Human Neurosciences, Policlinico Umberto I, Sapienza University of Rome, 00161 Rome, Italy; alexiakoukopoulos@gmail.com (A.E.K.); lorenzo.tarsitani@uniroma1.it (L.T.)

**Keywords:** post-traumatic stress disorder, PTSD, COVID-19, healthcare worker, risk assessment, risk management

## Abstract

Prevention of post-traumatic stress symptoms (PTSS) in healthcare workers (HCWs) facing the current COVID-19 pandemic is a challenge worldwide as HCWs are likely to experience acute and chronic, often unpredictable, occupational stressors leading to PTSS. This review aims to analyze the literature to discover which topics have been focused on and what the latest developments are in managing the occupational risk of PTSS in HCWs during the current pandemic. For the purpose of this review, we searched for publications in MEDLINE/Pubmed using selected keywords. The articles were reviewed and categorized into one or more of the following categories based on their subject matter: risk assessment, risk management, occurrence rates. A total of 16 publications matched our inclusion criteria. The topics discussed were: “Risk Assessment”, “Occurrence Rates”, and “Risk Management”. Young age, low work experience, female gender, heavy workload, working in unsafe settings, and lack of training and social support were found to be predictors of PTSS. This review’s findings showed the need for urgent interventions aimed at protecting HCWs from the psychological impact of traumatic events related to the pandemic and leading to PTSS; healthcare policies need to consider preventive and management strategies toward PTSS, and the related psychic sequelae, in HCWs.

## 1. Introduction

Minimizing the psychological impact of the current coronavirus disease 2019 (COVID-19) pandemic on healthcare workers (HCWs) represents a special challenge for healthcare systems through the world. In fact, HCWs represent the first-line fighters treating patients with COVID-19, and every day, they face a high risk of being infected and, consequently, of spreading the virus to other people [1,2,3]. HCWs are thus facing critical situations that increase their risk of suffering from the psychological impact of dealing with several unfavorable conditions, with consequences that might extend from psychological distress to mental health symptoms [4]. A body of evidence highlights that past infectious disease outbreaks, including the severe acute respiratory syndrome (SARS), the Middle East respiratory syndrome (MERS), and the 2009 novel influenza A (H1N1), were associated with mental health issues among HCWs [5,6], mostly post-traumatic stress symptoms (PTSS) and post-traumatic stress disorder (PTSD). In particular, research by Xiao et al. [7] revealed that HCWs working in settings exposed to a high risk of SARS were 2–3 times more likely to have high PTSS levels than those not exposed. Therefore, consistent with Dutheil et al. [8], the ongoing pandemic of COVID-19 is highly likely to also promote stress disorders in HCWs, potentially degenerating into chronic PTSD, as has already occurred in past outbreaks.

According to ICD-10, PTSD typically involves symptoms that can be classified into three groups: (1) intrusion—recurrent images, dreams or memories related to the traumatic experience; (2) avoidance—of places, people or topics related to the traumatic experience, accompanied by a general decrease in activity; (3) arousal—understood as increased psychophysiological reactivity in the form of attention-deficit disorders, circadian rhythm disorders, or increased vigilance [9]. In 2013, the DSM-5 [10] encoded important changes for what concerns post-traumatic stress conditions, particularly PTSD. In addition to changes in the symptomatologic diagnostic criteria, the current edition of the DSM better specified Criterion A about the trauma, eliminating the need of q person’s response to the event involving intense fear, helplessness, or horror (criterion A2) and better clarifying the characteristics of the potentially traumatic experiences including, for the first time, a repeated or extreme indirect exposure to aversive details of the event(s), usually in the course of professional duties (criterion A4). Given the current concern with the COVID-19 pandemic, a summary of the evidence is required to allow policy makers to enact guidance for protecting HCWs. In urgent circumstances such as the ongoing pandemic, rapid reviews are recommended by the WHO [11]. We conducted a review of the literature on PTSS in HCWs employed in hospital settings during the COVID-19 pandemic. The aims of the critical revision were to evaluate the related (1) risk assessment, (2) risk management, and (3) occurrence rate.

## 2. Materials and Methods

### 2.1. Search Strategy

We conducted a systematic review of literature from February 2020 to 15 October 2020, regarding PTSS in HCWs employed in hospital settings. The general methods and selection criteria were based on different sources. First, two major scientific databases (MEDLINE/Pubmed) were used; second, the reference sections of the identified studies were scanned for additional relevant studies satisfying the criteria. Selected keywords were used to identify articles for this review of literature. The keywords were: “post-traumatic stress disorder”, “PTSD”, “post-traumatic stress symptoms”, “PTSS”, “COVID-19”, “Sars-CoV-2”, “healthcare worker”, “hospital”, “assessment”, “management”, “occurrence”, and “prevalence”. The keywords were systematically combined in order to conduct the literature search. For example, “PTSD” AND “healthcare 2orker” AND “hospital” was one combination. We aimed to identify original articles (i.e., non-reviews) using the abovementioned keywords with the following exclusion criteria: (1) not written in English; (2) studies focused on PTSS not related to the COVID-19 pandemic; and (3) qualitative studies.

### 2.2. Data Extraction

The screening of articles was carried out in two phases. In the first phase, articles were screened on the basis of title and abstract. The abstracts of all the selected titles were sorted for more detailed information. Two independent reviewers (G.d’E. and P.V.) read the abstracts and categorized them as relevant, not relevant, or possibly relevant. In the second phase, the full-text articles were assessed for eligibility. Two reviewers (G.d’E. and P.V.) independently applied inclusion and exclusion criteria to potentially eligible papers and both reviewers then independently extracted data from the original articles. Any disagreements were independently checked by a third reviewer (G.C.) and a consensus was reached. The Newcastle–Ottawa scale (NOS) for observational studies was used to evaluate the quality of the studies [12].

### 2.3. Categorization of Selected Articles

Every full-text article that met the inclusion criteria was reviewed and categorized into one or more of the following three categories based on its subject matter: risk assessment (articles aimed at the identification of occupational risk factors for PTSS), risk management (articles focused on occupational interventions for reducing the likelihood of PTSS occurrence), and occurrence rates (e.g., incidence or prevalence of PTSS among HCWs). This systematic review was reported in accordance with the Preferred Reporting Items for Systematic Reviews and Meta-Analyses (PRISMA) statement [13].

## 3. Results

**Figure 1:**
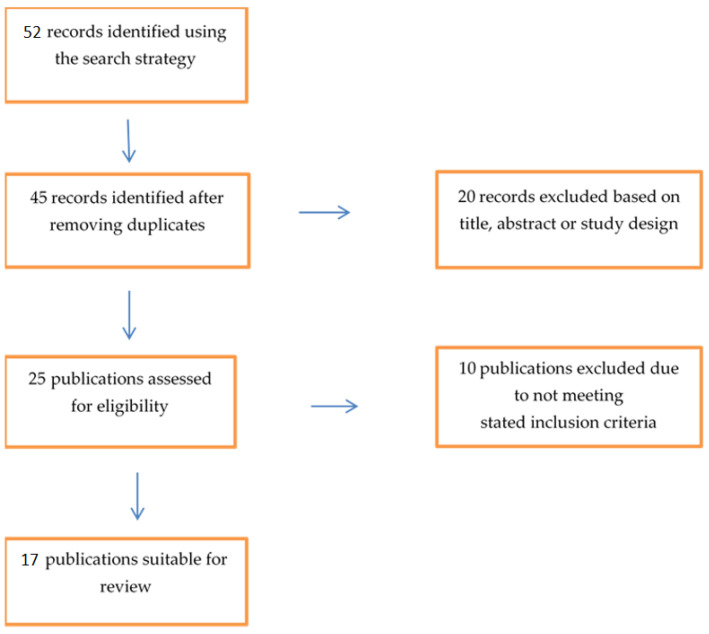
Study flow chart. The flow chart summarizes how the articles were reviewed and categorized based on risk assessment, risk management, and occurrence rates topics.

The initial search retrieved 52 articles that matched our inclusion criteria. After reviewing the titles and abstracts, 35 articles were excluded. Therefore, 17 papers remained in the study (Figure 1). These 17 papers were then categorized according to their subject matter. The topics, discussed in order of frequency from highest to lowest, were risk assessment, occurrence rates, and risk management. All 17 papers assessed the risk of PTSS; 14 articles focused on occurrence rates, and 5 articles on risk management. In total, 3 papers targeted all three topics, 14 papers studied both risk assessment and occurrence rates, and 1 paper analyzed only risk assessment of PTSS (Table 1). Characteristics, outcomes, and main results of the selected studies are presented in Table 2.

## 4. Discussion

During the current COVID-19 pandemic, HCWs face unprecedented scenarios often outside their ordinary levels of experience and training, as they are at the forefront of the fight against the virus worldwide. This critical situation increases HCWs’ risk of suffering from symptoms ranging from psychological distress to psychiatric disorders, as a result of the effort to continuously fight with several COVID-related unfavorable conditions [4]. To better clarify the characteristics of the pandemic-related traumatic experiences, we conducted a review of literature on PTSS in HCWs employed in hospital settings during the COVID-19 pandemic, focusing on risk assessment, risk management, and occurrence rates.

### 4.1. Risk Assessment of Work-Related PTSS

The 17 articles focusing on the risk assessment of work-related PTSS among hospital HCWs during the COVID-19 pandemic aimed to identify the risk profile for HCWs with regard to organizational and individual factors making HCWs susceptible to PTSS. In particular, 11 papers analyzed the pre-trauma risk factors and detected predictors of PTSS at individual level, 5 articles focused on post-traumatic risk factors of PTSS at both organizational and individual level, and 3 papers focused on both the pre- and post-traumatic risk factors. Regarding the individual pre-trauma risk factors, female gender, young age, low work experience, not living with a partner, and lack of training were found to be related to a high risk of PTSS. Most of the selected articles showed female gender at a higher risk than male, consistent with existing studies on PTSS in the general population and in HCWs before the COVID-19 outbreak [30,31]; given this gender susceptibility to PTSS, Gonzalez-Sanguino et al. [26] hypothesized that during the current pandemic, women tend to take on a caregiving role at home and, because of the need to balance this with healthcare work, are consequently at increased risk and more vulnerable in this situation of overload. By contrast, Song et al. [19], in a cross-sectional study conducted in China, found increased occurrence of PTSS in male medical staff compared to female and concluded that this difference may be due to the timing of their study, which was conducted later than the comparative studies, when the psychological impact of stressors may have been minimized and the mental status of female medical staff may have gradually improved over time. Moreover, women tend to pay gradually more attention to their experiences and feelings and are more willing to express their emotions; this behavior leads to the self-regulation of emotions and, therefore, may defuse the impact of stressors over time [16,32]. A convergence was found on the increased risk of PTSS in medical staff who are unmarried, divorced, or widowed; based on this finding, Song et al. [19] speculated that such HCWs receive less care and/or communication from their partner and may experience less family support, as highlighted by other studies that showed increased risk of PTSS in workers lacking social support.

Interestingly, a relationship was found between lack of medical training on COVID-19 and occurrence of PTSS; in fact, increased rates of PTSS were found among untrained HCWs than among trained ones; in particular, a multicentric study performed by Chew et al. [16] found that non-medically trained HCWs were at higher risk of adverse psychological outcomes, including PTSS, compared to their medically trained counterparts. This is in agreement with a recent study on HCWs during the start of the pandemic in Singapore [17], as well as a recent study in China [16] that surprisingly demonstrated that frontline nurses had significantly decreased vicarious traumatization scores in comparison with non-frontline nurses during the COVID-19 pandemic; moreover, a relationship was found between the lack of accessibility to first-hand medical information on the pandemic, less formal training and confidence in infectious control measures, and increased occurrence of traumatization in non-frontline and non-trained nurses compared to frontline and trained nurses [17].

Younger age was found to be a risk factor for anxiety and PTSS, in accordance with data regarding workers different from HCWs; in particular, past studies found that in the general population, older adults have better mental health than younger adults [33,34], and this seems to hold true during the current pandemic [14]. It is also possible that older HCWs are more experienced and better equipped both professionally and psychologically to deal with the stress of the pandemic. Consistent with this hypothesis, an Italian report [35] aimed at the organizational changes and management challenges presented by the pandemic found that the difficulties in patient care were likely greatest for the newer, lesser-skilled nurses. These results lead us to conclude that nurse managers should increase training and support related to COVID-19 care for younger, less experienced nurses in order to mitigate mental health problems, including PTSS [14].

Post-traumatic risk factors of PTSS were found as follows: low social support at work, heavy workload, working in unsafe settings (e.g., lack of personal protective equipment), passive coping, anxiety, and burnout. Considering that the healthcare system has operated under the most challenging conditions during the period of pandemic, the related emotional and physical exhaustion in HCWs represents a major concern. Work-related consequences of this condition, called burnout, include poorer quality of care, professional mistakes, probably reduced attention to individual protection procedures, and increased risk of contagion in the workplace [36]. The impacts of burnout on the safety of HCWs and patients highlight the need for early identification of this health condition in the work environment and preventive interventions. In particular, management should be proactive in and supportive of improving working conditions to avoid burnout risks: implementation of training and technical updates on COVID-19 for HCWs, adequate supply of personal protective equipment (PPE), ban of prolonged working hours, and availability of counseling services have been proposed as possible responses [18,36].

This present review found strong concordance regarding the negative relationship between social support and PTSS. According to the literature, lack of social support is a known predictor of occupational stress in HCWs [37,38], and it is defined as “the feeling that one is cared for and has assistance available from other people” and “that one is part of a supportive social network” [38]. As revealed by Si et al. [21], a supportive work environment is a buffering factor of negative psychological health among HCWs and protects them from PTSS during the COVID-19 pandemic; in fact, in their study, the authors found that social support had the greatest impact on the mental health of HCWs: low (OR: 5.49, 95% CI: 4.04, 7.45) and moderate (OR: 2.73, 95% CI: 2.42, 3.07) levels of social support were associated with a higher risk of PTSD compared to high levels of social support [19].

During the first wave of COVID-19, the lack of personal protective equipment (PPE) was identified as a top concern for HCWs throughout the world, and results of the current review indicate that this was a significant factor impacting HCWs’ mental health; in fact, in a cross-sectional study, Arnetz et al. [14] found a reverse dose–response relationship between lack of PPE provision and PTSS scores: the severity of PTSS was significantly lower as PPE provision frequency increased (PTSS scores decreased from 14.0 (9.0–18.0) to 9.0 (7.0–13.0), *p* < 0.001); moreover, working in potentially lethal settings but lacking PPE was found to be a worse factor impacting the mental health of HCWs and leading to depression and anxiety other than PTSS. Actually, PPI shortages are still a real problem for many health professionals, especially in areas with less prosperous economies or poor health services. The availability of effective barrier devices whose quality inspires trust is crucial to reduce the perception of danger of HCVs in the setting of transmissible diseases. In fact, even more than preventive behaviors, barrier devices are perceived as an extension of the body’s ability to defend itself against external aggression, a sort of shield or armor able to physically stop the virus; in this sense, their continued availability is essential to fueling the resilience of HCWs [39].

### 4.2. Risk Management of PTSS

The articles focused on risk management showed the ways to moderate the occurrence of PTSS through HCWs’ occupational training and the improvement of social support at work. Convergence was found about the need of occupational training targeted at pandemic and infectious control measures. Interestingly, Chew et al. [16] observed that passive training through educational pamphlets, emails, or websites was effective in minimizing PTSS in HCWs experiencing psychological distress. In particular, education on the natural history of the virus and the appropriate use of infection control measures, especially for non-medically trained HCWs, was a predictor of lower risk of PTSS compared to that for non-trained HCWs who received no such education. Moreover, brief passive psychoeducational interventions targeting high-risk groups were shown to be effective during the peak of the pandemic, although to date, the extent of their benefit remains unknown. Consistent with these studies, Caillet et al. [28] showed lack of medical training as an independent factor for PTSS in HCWs employed in COVID-19-intensive care units (OR = 2.155 (95% CI, 1.047–4.440), *p* = 0.04); given this finding, the authors suggested the need for training and re-training HCWs continuously exposed to the risk of both acquiring the disease and spreading SARS-CoV-2 to their family, friends, and colleagues. Regarding the protective role of training, interesting research was conducted by Tan et al. [17] evaluating, among others, traumatic stress among medical workers (doctors and nurses) and non-medical workers (caregivers, pharmacists, technicians. administration, office workers, and cleaners) in Singapore. The authors observed a lower mean PTSS score among HCWs during the COVID-19 pandemic than during the SARS epidemic, when the severity of PTSS was three times higher than in the current pandemic. These findings led the authors to infer that respondents were more prepared for the COVID-19 pandemic, and they implemented infection control procedures following experiences from the previous SARS epidemic.

A body of evidence has shown that training should also be targeted at improving a supportive work environment. Therefore, according to existing literature, hospital management and supervisors need to anticipate the effects of traumatic exposure by training HCWs in evidence-based anticipatory methods of coping with stressful events, in reducing the development of post-traumatic stress reactions and general distress, and in educating HCWs to support their colleagues after adverse events [40]. A study by Zhang et al. [3] showed that simple standardized questionnaires can be used, which should be supplemented with a brief questionnaire assessing work conditions (demands, decision latitude, and support). A description of a protective program has been published by Cao et al. [41] based upon hands-on recent experience in China. An important part of that program was the repeated use of a short questionnaire supplemented with personal interviews with a representative group of employees.

Moreover, according to Theorell [42], a supportive leadership should implement the following measures to minimize the impact of stressors on the psychological status of HCWs dealing with the current pandemic: (1) flexible work schedules that are adapted to the ever-changing situation; (2) sleep hygiene, which is facilitated by wise shift cycles and good possibilities for undisturbed sleep; (3) social support to family members; worries for family members could add to the caregiver’s health deterioration; (4) participation in decision making; (5) facilitation of good coping mechanisms; (6) facilitation of cultural experiences, for instance, easy electronic access to films, concerts, and lectures during leisure time. Supervisors should also be involved in monitoring the health of their staff. Finally, with regard to the coping strategies adopted by HCWs to minimize their stress levels, Vagni et al. [24], in a cross-sectional study, showed that blocking negative or unpleasant emotions and thoughts reduced the arousal and intrusion levels of the trauma during the emergency phase of the pandemic, unlike problem-focused strategies. Based on this finding, the authors hypothesized that HCWs, during the ongoing COVID-19 pandemic, frequently incur a lack of a cognitive process of emotions, thus failing to identify their emotional reactions, which tends to be associated with maladaptive behaviors leading to increased risk of PTSS. Moreover, problem-focused coping strategies were not found to be effective in protecting HCWs in the first wave of the pandemic, probably due to a lack of scientific knowledge about therapeutic and treatment procedures effective for COVID-19.

### 4.3. Occurrence of PTSS

The 14 selected articles analyzing the occurrence of PTSS in HCWs dealing with the COVID-19 pandemic showed a range between 2.1% and 73.4%. This wide range is attributable to (1) the different timing of the studies and (2) the healthcare settings investigated. In particular, Song et al. [19], in a study conducted from 28 February 2020 to 18 March 2020, when the outbreak in China had been controlled and the work pressure of the HCWs was significantly reduced compared to the peak period of the outbreak, found lower prevalence rates of PTSS (9.1%) compared to studies conducted during the peak period of COVID-19 in China [43]. In line with this finding, the study by Rossi et al. [25], performed in Italy between 27 and 31 March, during the peak of the outbreak in Italy, showed a higher occurrence of HCWs being affected by PTSS, compared to studies conducted later, when the pandemic was controlled. Regarding healthcare settings, increased rates of PTSS were found in inpatient settings (up to 70%), particularly among HCWs caring for COVID-19 patients or employed in emergency hospital wards [14].

### 4.4. A Call for Action

The findings of the present review show the need for urgent interventions aiming to protect HCWs from the psychological impact of traumatic events related to the pandemic and leading to PTSS. A body of evidence has been found about the following predictors of increased risk of suffering from PTSS: young age, female gender, not being a graduate, heavy workload, low medical training, not living with a partner, and low social support. Therefore, healthcare organizations should focus on the supportive resources available for HCWs in order to prevent the heavy psychological impairment related to PTSS in HCWs dealing with the COVID-19 outbreak. In fact, PTSS involve chronic severe anxiety with re-experiencing of the traumatic event, flashbacks, nightmares, increased arousal, and reduced social life and could lead to PTSD. As people suffering from PTSD are more at risk of suicidal ideation, suicide attempts, and death by suicide in huge proportions (2–5 times) [44], the prevention of PTSD is a special concern for HCWs, considering that they are already in occupations which are at increased risk [45], and people suffering from PTSS are prone to not seeking care because of barriers such as lack of information, being afraid of stigmatization, or the belief that symptoms may decrease with time [46]. Presently, a special effort is required to prevent PTSD as a secondary effect of the SARS-Cov-2 pandemic among HCWs facing COVID-19 patients. Therefore, healthcare policies need to take into account preventive and management strategies toward PTSS, and the related psychic sequelae, as soon as possible, including policies to implement regular screening of PTSS in HCWs. There is also a need for urgent intervention to identify and treat HCWs with PTSD, as such an approach can reduce the risk of chronic psychological impairment.

## 5. Limits of the Study

There were several limitations in this review. Firstly, because of the use of the inclusion criteria, we could have missed potentially relevant papers in the first step of data selection. Secondly, the cross-sectional profile of all the selected studies limits the possibility of drawing strong conclusions; therefore, caution should be taken in generalizing the findings. Moreover, the findings could have been influenced by organizational factors intrinsic to the local occupational context of each study and, consequently, not be true for all healthcare workers: in particular, the different (a) geographical contexts, (b) cultural variables, and (c) chronological periods of the COVID-19 pandemic in which the aforementioned studies were carried out may have influenced the different types of psychological responses to the same stressor among HCWs. Interestingly, the totality of the selected works was produced mainly in South East Asia and China and in Western Europe, and only two in the United States and Mexico. This evidence can obviously constitute a bias and somehow orient the reading of the topic on the basis of specific cultural backgrounds. Finally, the wide range of occurrence rates observed is attributable to different (1) healthcare settings investigated and (2) timing of the studies; for this reason, a direct comparison between the data provided by the different studies is not always possible and can in some cases be misleading.

## 6. Conclusions

The SARS-CoV2 pandemic represented a challenge at multiple levels: for the management of public health, for the discovery of new therapeutic and vaccine resources, for the understanding of etiology and pathogenesis [47,48,49,50,51,52]. However, it also posed a great challenge for healthcare workers forced to measure themselves against a disease that was risky for their own health as well as that of their patients. This systematic review of the literature showed young age, low work experience, female gender, heavy workload, working in unsafe settings, and lack of training and social support as predictors of PTSS. Moreover, the need for urgent interventions aimed at protecting HCWs from the psychological impact of traumatic events related to the pandemic and leading to PTSS is increasingly a key issue in the management of COVID-19 pandemic. Finally, there is an urgent need to define new healthcare policies devoted to preventive and management strategies toward PTSS, and the related psychic sequelae, in HCWs.

## Figures and Tables

**Table 1:** Summary of the studies included in the review.

Reference	Study Design	Study Location		Sample Size	Risk Assessment	Risk Management	Occurrence Rates	Measures	**Quality Assessment NOS Score**
Arnetz et al. [14]	Cross sectional	America	United States	695	X		X	PCL-6 (*)	7
Ramirez et al. [15]	Cross sectional		Mexico	3932	X		X	IES-R (**)	6
Chew et al. [16]	Cross sectional	Asia	India Singapore Indonesia Malaysia Vietnama	384 277 250 175 60	X	X	X	IES-R (**)	5
Tan et al. [17]	Cross sectional		Singapore	470	X		X	IES-R (**)	5
Khasne et al. [18]	Cross sectional		India	2026	X		X	CBI (***)	
Ying et al. [2]	Cross sectional		China	371	X		X	PCL-5 (****)	5
Song et al. [19]	Cross sectional		China	13.879	X	X	X	PCL-5 (****)	7
Wang XY et al. [20]	Cross sectional		China	202	X		X	PTSD Checklist-Civilian Version	6
Si et al. [21]	Cross sectional		China	863	X		X	IES-6 (*)	6
Wang Y et al. [22]	Cross sectional		China	1257	X		X	IES-R (**)	7
Asaoka et al. [23]	Cross sectional		Japan	331	X			IES-R (**)	4
Vagni et al. [24]	Cross sectional	Europe	Italy	210	X	X		STSS-I (*****)	7
Rossi et al. [25]	Cross sectional		Italy	1379	X		X	Italian version of the Global Psychotrauma Screen (GPS)	
Gonzàlez-Sanguino et al. [26]	Cross-sectional		Spain	3480	X		X	PCL-C-2 (******)	7
Luceno-Moreno et al. [27]	Cross sectional		Spain	1422	X		X	IES-R (**)	6
Caillet et al. [28]	Cross sectional		French	208	X	X	X	IES-R (**)	7
Nowicki et al. [29]	Cross sectional		Poland	325	X	X		IES-R (**)	7

**Legend:** (*) 6-item version of the full 20-item post-traumatic stress disorder (PTSD) checklist; (**) Impact of Event Scale—Revised; (***) Copenhagen Burnout Inventory; (****) PTSD checklist for DSM-5; (*****) Secondary Traumatic Stress Scale—Italian version; (******) civilian version of the Post-traumatic Stress Disorder Checklist—Reduced version.

**Table 2:** Characteristics, outcomes, and main results of the studies included in the review.

Geo-Graphical area	Reference	Primary Outcome	Methods/Investigation Period/Setting	Occurrence Rate/Main Results/Risk Management
**North and Central America**	Arnetz et al. [14]	To evaluate the association between access to adequate PPE and mental health outcomes among U.S. nurses	Online questionnaire/May 2020/COVID-19: nurses	symptoms of depression 59.5% (severe depression 9.7%) anxiety 54.9% (severe anxiety 8.3%) symptoms of PTSD 29.1% Subjects lacking access to adequate PPE reported a higher impact of symptoms of depression, anxiety, and post-traumatic stress disorder
	Ramirez et al. [15]	To evaluate the presence of psychological distress, signs of post-traumatic stress, and to identify the groups of subjects at highest risk	Online survey/March–April 2020/COVID-19: volunteers, mainly students and employees of a Mexican University, HCWs and their contacts	Post-traumatic stress 27.7% Psychological distress: ○ intrusive thoughts 22% ○ avoidance 22.3% ○ hyperarousal 12.2% Younger ages, female sex, employed status, single condition, socially isolating, more days in isolation, a perception of a high risk of contracting COVID-19, a change in routine, less activity and having been economically impacted were variables positively correlated with higher psychological distress.
**South-East Asia**	Chew et al. [16]	To compare the psychological outcomes during the COVID-19 pandemic and identify factors associated with adverse psychological outcomes	Self-administered survey/April–June 2020/COVID-19: HCWs	Ranges in analyzed countries: Depression from 0,8% to 14,3% Anxiety from 0,8% to 6,7% Stress from 3,3% to 6,8% PTSD from 2,1% to 15% Non-medically trained personnel, the presence of physical symptoms, and presence of prior medical conditions were independent predictors of adverse psychological outcome. *Resources proposed to reduce PTSS:* ❖ Improving accessibility to early psychological support (in the form of counselling, internet-based cognitive–behavioral therapy) ❖ Passive psychoeducation (educational pamphlets, emails or website)
	Tan et al. [17]	To examine the psychological distress, depression, anxiety, and stress experienced by healthcare workers	Self-administered survey/February–March 2020/COVID-19: HCWs	anxiety 14.5% depression 8.9% stress 6.6% clinical concern of PTSD 7.7% The prevalence of anxiety was higher among non-medical healthcare workers than medical personnel. Non-medical HCWs are at highest risk for psychological distress.
	Khasne et al. [18]	To evaluate the prevalence of burnout in HCWs involved in the care of COVID-19 patients	Questionnaire-based survey/date not reported/COVID-19: HCWs	personal burnout 44.6% work-related burn-out 26.9% pandemic-related burnout 52.8% Pandemic-related burnout was 1.67 times more frequent in medical doctors and 5 times in support staff. The prevalence of personal and work-related burnout was significantly higher among younger respondents and females.
	Ying et al. [2]	To investigate the mental health status and related factors in families of HCWs employed in hospital	Online self-administered questionnaires/February 2020/COVID-19: families of HCWs	anxiety 33.73% depression symptoms 29.35% Risk factors for depressive symptoms were more time spent thinking about COVID-19, longer average working time per week worked by family members (that is, HCWs), and being parents and other next of kin of HCWs.
	Song et al. [19]	To assess the mental health of emergency department medical staff during the SARS-CoV-2 epidemic	Electronic questionnaires/February–March 2020/COVID-19: HCWs	Depressive symptoms 25.2% PTSD 9.1% A higher risk of developing depressive symptoms and PTSD was present in HCWs who were middle-aged, who had worked for fewer years, had longer daily work time, and had lower levels of social support. Being a man was associated with a higher probability of depressive symptoms and PTSD. Nurses had a higher risk of PTSD than doctors. *Resources proposed to reduce PTSS:* ❖ Psychological skills training to better regulate the psychological status of medical staff and to mitigate the psychological problems ❖ Targeted psychological interventions to promote the mental health of medical staff
	Wang XY et al. [20]	To investigate the factors potentially involved in the level of PTSD of nurses exposed to COVID-19	Questionnaires/February–March 2020/COVID-19: Nurses	PTSD 16.83% Job satisfaction and gender were influencing factors of PTSD. PTSD was negatively correlated with positive coping, and positively correlated with negative coping.
	Si et al. [21]	To identify and evaluate the psychological impact of COVID-19 on medical care workers	Questionnaires/February–March 2020/COVID-19: medical care workers	Post-traumatic stress (PTS) disorder symptoms 40.2% symptoms of ○ depression 13.6% ○ anxiety 13.9 % ○ stress 8.6% Perceived threat and passive coping strategies were positively correlated with PTS and depression, anxiety, and stress. Perceived social support and active coping strategies were negatively correlated to depression, anxiety, and stress.
	Wang Y et al. [22]	To evaluate the acute psychological effects of COVID-19 outbreak among HCWs	Survey/Early period of pandemic in China/COVID-19: HCWs	depression 15.0% anxiety 27.1% PTSD 9.8% Risk factors for depression and anxiety were having an intermediate technical title, working at the frontline, receiving insufficient training for protection, and lacking confidence in protection measures. Risk factors for PTSD were being a nurse, having an intermediate technical title, working at the frontline, and lacking confidence in protection measures. Protective factor for developing depression, anxiety, and PTSD was not worrying about infection.
	Asaoka et al. [23]	To evaluate factors associated with post-traumatic stress symptoms (PTSS) among HCWs highly involved in COVID-19-related activities outside hospitals	Internet-based survey/March–April 2020/COVID-19: HCWs	Anxiety about infection, exhaustion and being a Disaster Psychiatric Assistance Team member were associated with PTSS.
**Western Europe**	Vagni et al. [24]	To explore the relationship between coping strategies used by HCWs and emergency workers to manage the stress factors related to the SARSC-CoV-2 epidemic	Questionnaires online/date not reported/COVID-19: HCWs and emergency workers	HCWs had greater levels of emergency stress and arousal and were more willing to use problem-focused coping compared with emergency workers. HCWs are exposed to a large degree of stress and could experience secondary trauma linked with their involvement in the treatment of SARS-CoV-2 infection. *Resources proposed to reduce PTSS:* ❖ Improving individual efficacy in stopping negative thoughts and emotions.
	Rossi et al. [25]	To evaluate mental health outcomes among HCWs	Online questionnaire/March 2020/COVID-19: HCWs	PTSS 49.38 % Symptoms of depression 24.73% symptoms of anxiety 19.80% insomnia 8.27% high perceived stress 21.90% Younger age and female sex were associated with all investigated outcomes except insomnia. Being a frontline HCW or a general practitioner was associated with PTSS. Having a colleague who died was associated with PTSS, depression, and insomnia.
	Gonzàlez-Sanguino et al. [26]	To evaluate the psychological impact of the COVID-19 outbreak in a sample of the Spanish population	Survey/Early period of the pandemic in Spain/COVID-19: Spanish population and HCWs	depressive symptoms 18.7% anxiety 21.6% PTSD symptoms 15.8% Factors positively related to depression, anxiety, and PTSD: female gender, previous diagnoses of mental health problems or neurological disorders, loneliness, having symptoms associated with the virus, or having a close relative infected. Factors negatively related to depression, anxiety, and PTSD: Being in the older age group, having economic stability, and the belief that adequate information had been provided about the pandemic.
	Luceno-Moreno et al. [27]	To analyze post-traumatic stress, anxiety, and depression during the SARS-CoV-2 pandemic To evaluate associations between COVID-19 and burnout, resilience, work	Online survey/April 2020/COVID-19: HCWs	post-traumatic stress disorder 56.6%, anxiety disorder 58.6% depressive disorder 46% feeling of emotional emptying 41.1% The risk variables for anxiety and depression: female sex, working 12/24-h shifts, and being worried that a family member could be infected.
	Caillet et al. [28]	To assess the psychological impact of COVID-19 on ICU caregivers at the peak of the “crisis period”	Survey/April 2020/COVID-19: ICU HCWs	anxiety 48% depression 16% PTSD symptoms 27% Risk factors for: ○ anxiety syndrome: not being trained in intensive care medicine, being assigned in COVID-19 ICU. ○ PTSD: having a history of burn-out, not being trained in ICU. *Resources proposed to reduce PTSS:* ❖ informing ICU HCWs about the SARS-CoV-2 outbreak (route of transmission and prevention procedures) ❖ Implementing prevention procedures (ICU training sessions) in persons at risk.
	Nowicki et al. [29]	To investigates the level of post-traumatic stress, perceived social support, sense of security, and sense of meaning among nurses in the face of the SARS-CoV-2 epidemic.	Computer-assisted web interviews/May 2020/COVID-19: Nurses	An intensification of traumatic stress symptoms with particularly pronounced symptoms of avoidance was observed in subjects evaluated. Nurses characterized the experience of a pandemic with a reduced sense of security and an intense reflection on issues related to their personal safety. *Resources proposed to reduce PTSS:* ❖ individual support delivered to HCWs mostly from significant subjects, other than friends and family. ❖ Underlining positive changes resulting from experiences related to the SARS-CoV-2 pandemic. ❖ Enhance adaptation in the form of post-traumatic growth.
